# Odorant ligands for the CO_2_ receptor in two Anopheles vectors of malaria

**DOI:** 10.1038/s41598-019-39099-0

**Published:** 2019-02-22

**Authors:** Iliano V. Coutinho-Abreu, Kavita Sharma, Liwang Cui, Guiyun Yan, Anandasankar Ray

**Affiliations:** 1 Department of Molecular Cell and Systems Biology, University of California Riverside, Riverside, CA 92521 USA; 2 Department of Entomology, The Pennsylvania State University, University Park, Pennsylvania 16802 USA; 3 Public Health and Ecology & Evolution Biology, University of California, Irvine, CA 92697 USA

## Abstract

Exhaled CO_2_ is an important host-seeking cue for *Anopheles* mosquitoes, which is detected by a highly conserved heteromeric receptor consisting of three 7-transmembrane proteins Gr22, Gr23, and Gr24. The CO_2_ receptor neuron has been shown to also respond sensitively to a variety of odorants in *Aedes aegypti*. The detection of CO_2_ is important for upwind navigation and for enhancing the attraction to body heat as well as to skin odorants. The orthologs of the CO_2_ receptor proteins are present in malaria-transmitting mosquitoes like *Anopheles coluzzii* and *Anopheles sinensis*. Activators and inhibitors of the CO_2_-neuron were tested on the maxillary palps in these two species by single-sensillum electrophysiology. The electrophysiological testing of three prolonged-activator odorants identified originally in *Aedes aegypti* also showed varying ability to reduce the CO_2_-ellicited increase in spikes. These findings provide a foundation for comparing the functional conservation with the evolutionary conservation of an important class of odorant receptor. The identification of a suite of natural odorants that can be used to modify the CO_2_-detection pathway may also contribute to odor-blends that can alter the behavior of these disease transmitting mosquitoes.

## Introduction

Vector-borne diseases cause significant morbidity and mortality throughout the globe. Mosquitoes transmit a variety of pathogenic microorganisms that are responsible for malaria, filariasis, dengue fever, and encephalitis. Anopheline mosquitoes transmit malarial parasites, which infect >216 million people in Africa and Asia, resulting in ~445,000 deaths annually^1^. Indoor residual insecticide spraying (IRS) and long-lasting insecticide treated bednets (LLINs) provide protection against endophilic/endophagic mosquitoes, but lack of protection from bites for activities outside a bednet or outdoors from exophilic and diurnal mosquitoes require the development of new tools.

Mosquitoes are attracted long-range to carbon dioxide (CO_2_) exhaled from human breath and short-range to skin odor and body temperature^2,3^. The heteromeric CO_2_-receptor is expressed in the capitate peg sensillum A neuron (cpA) on the maxillary palps and consists of three 7-transmembrane proteins of the Gustatory Receptor family (Gr22, Gr23, and Gr24)^4,5^. This heteromeric receptor also detects skin odorants in *Aedes aegypti* and *Anopheles coluzzii*, participating in attraction when tested in *A. aegypti*^6^. Several additional classes of odorants have been identified using chemical informatics and electrophysiology that either activate or inhibit the CO_2_ receptor neuron in *A. aegypti*^6^. Given the sequence-level conservation of the orthologous Gr receptors in anopheline species, it would be interesting to study whether or not the physiological responses to known ligands are also conserved. Activators of the CO_2_ receptor that we identified previously have been shown to lure mosquitoes into a trap when tested in a greenhouse for *Ae. aegypti* and in semi-field conditions for *An. coluzzii*^6,7^. Identification of additional activators of the CO_2_ receptor in anophelines could therefore assist in development of convenient synthetic odorant lures that can mimic CO_2_.

Detection of skin odorants also occurs through other receptors belonging to the Or and Ir families^8–10^, as a *Gr3* receptor mutant female *Ae. aegypti* can still seek out a human inside a greenhouse^11^. While these alternative pathways exist in mosquitoes to sense mammalian hosts, there is still a reduction in host-seeking behavior of *Ae. aegypti* by interfering with the detection of CO_2_ using inhibitory odorants^6^ or genetically when testing mice in a large cage arena^12^. The identification of odorant ligands of the *Anopheles* CO_2_ receptor neuron (cpA) can contribute to the design of attraction masking agents, which could reduce anopheline biting rates.

Using single sensillum recordings, we screened the cpA neuron of two anopheline mosquito species, anthropophilic *An. coluzzii* and facultative anthropophilic *Anopheles sinensis*, that transmit malaria with a large set of odorants that were initially identified by chemical-informatics as putative CO_2_ receptor ligands, and subsequently tested in the *Ae. aegypti* mosquitoes^6^. We identify several odorants that show conserved effects as activators, inhibitors, and ultra-prolonged activator of the cpA neuron. Some of these odorants have potential in reducing anopheline-biting rates.

## Results

### Sequence conservation of CO_2_ receptor proteins

**Figure 1:**
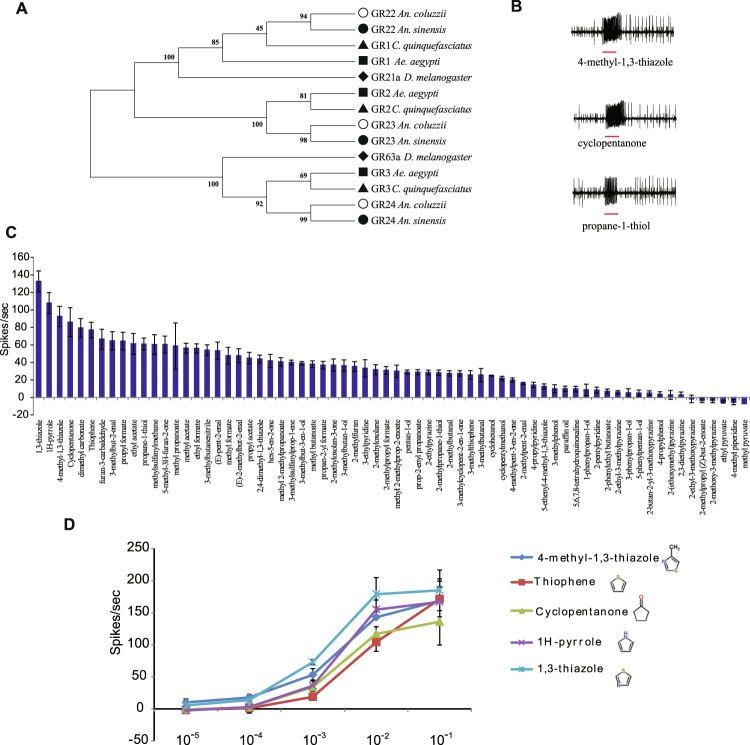
(**A**) Maximum likelihood tree depicts the phylogenetic relationship among the CO_2_ receptors GR22, GR23, and GR24 orthologs in mosquitoes. Mosquito species as labeled as follow: Open circles, *An. coluzzii*; filled circles, *An. sinensis*; filled squares, *Ae. aegypti*, filled triangles, *Cx. quinquefasciatus*; and filled diamond, *D. melanogaster*. Bootstrap support for the branches is also shown. (**B**) *An. coluzzii* cpA neuron representative traces depicting activation of the cpA neuron upon exposure to 0.5 second pulse (red horizontal bar) of the described odor and (**C**) mean responses to 68 chemical volatiles (headspace above 1% solution on filter paper). n = 4. error = s.e.m. (**D**) Dose-responses of the activators of the *An. coluzzii* cpA neuron by five strong activators and their chemical structures. N = 3. error = s.e.m.

There is a close relationship among CO_2_ receptors in the Anophelinae (*An. coluzzii* and *An. sinensis*) and Culicinae (*Ae. aegypti* and *Culex quinquefasciatus*) mosquitoes, and orthologs cluster together in distinct branches (Fig. 1A). The percent amino acid identities between the sequences of these mosquito orthologs ranged as follows: GR22 (81–90%), GR23 (83–97%), and GR24 (74–92%). Similarities ranged for GR22 from 88% to 95%, for GR23 between 94% and 98%, and for GR24 from 85% to 91%.

### Conservation of response to agonists in *An. coluzzii*

In order to test conservation of the CO_2_-receptor neuron responses to different odorants, we tested a structurally diverse set of 67 ligands of the CO_2_-receptor previously identified in *Ae. aegypti* and *Cx. quinquefasciatus*^6^. The *An*. *coluzzii* CO_2_ neuron responded to several of these odorants with different chemical structures (Fig. 1B). Out of the 67 odors tested 35 (52%) evoked responses ≥30 spikes/sec whereas 14 odors (21%) showed responses lower than the solvent (paraffin oil; Fig. 1C). The odorants that evoked the strongest activation from the *An. coluzzii* cpA neuron were further evaluated in dose-response assays across a range of five orders of magnitude. All odorants showed a dose-response and four out of the five odors still evoked responses ≥30 spikes/sec with headspace from a 10^−3^ dilution (Fig. 1D).

### Conservation of ultra-prolonged activators in *An. coluzzii*

**Figure 2:**
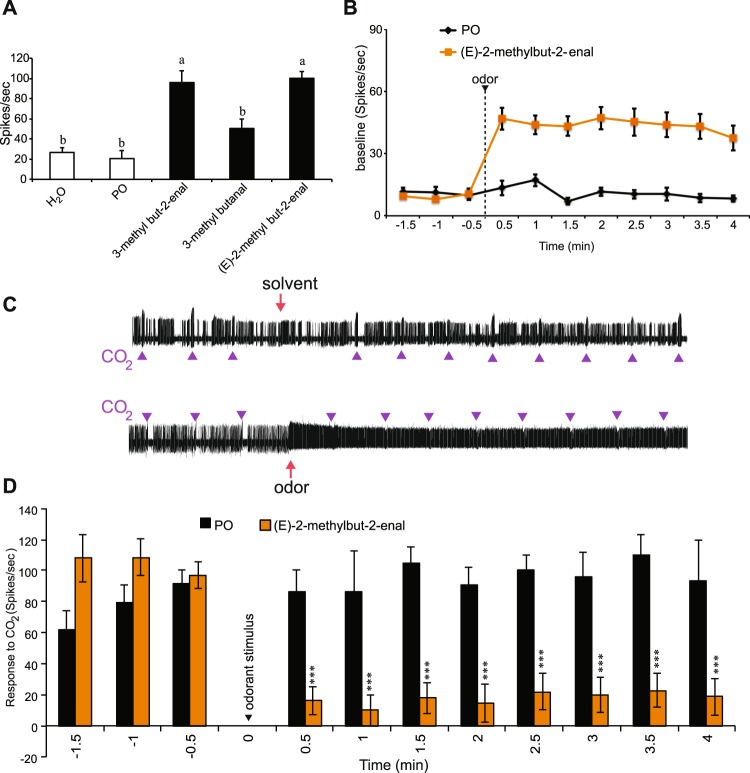
(**A**) Mean response of *An. coluzzii* cpA neuron in females to the ultraprolonged activators (headspace above 10% solution on filter paper) and the solvent (n = 5–6). **(B)** cpA baseline activity exposure to odorant. **(C**) Representative traces from the cp sensillum to 1 s pulses of 0.15% CO_2_ prior to and following a 3-s exposure to either solvent (PO-paraffin oil) or (E)-2-methylbut-2-enal (headspace above 10% solution on filter paper). (**D**) Mean responses of the cpA neuron to 1 s pulses of 0.15% CO_2_, calculated by subtracting 1-s of baseline activity prior to each stimulus after exposure to paraffin oil (gray) or (E)-2-methylbut-2-enal (headspace above 10% solution on filter paper) (orange). n = 5–6 individuals; t test, ***p < 0.001. Error = s.e.m.

Another class of ligands that have been identified in *A. aegypti* are ultraprolonged activators of the CO_2_ neuron^6,13^. In order to test these longer-term responses, recordings were performed as before with three known odorants (Fig. 2A). This analysis revealed that two of three odorants are conserved in their ability to evoke ultraprolonged activation in *An. coluzzii*. After a 3-s exposure to a (E)-2-methylbut-2-enal stimulus, the cpA neuron continues firing at ~50 spikes/sec for at least 5 min (Fig. 2B,C). Consistent with previous results responses to repeated CO_2_ stimuli during this 5-min period after the pre-exposure to (E)-2-methylbut-2-enal were significantly reduced (Fig. 2D).

### Conservation of inhibitors in *An. coluzzii*

**Figure 3:**
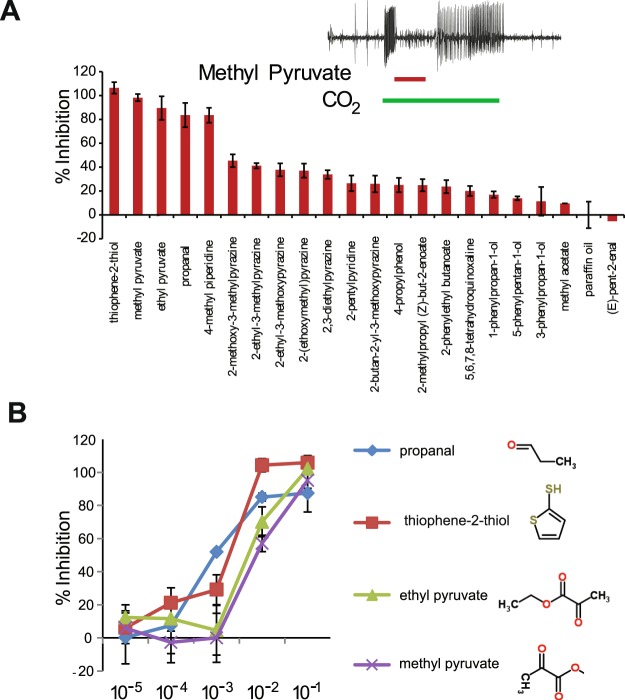
Inhibition of CO_2_ sensing by the *An. coluzzii* cpA neuron with specific chemical volatiles. **(A**) Overlays of CO_2_ and specific inhibitors can fully or partially inhibit the CO_2_- mediated activation of the cpA neuron. % Inhibition of CO_2_-evoked responses is measured in relation to exposure of the cpA neuron to CO_2_ and solvent. n = 4. Vertical bar represents s.e.m. Inset: Representative trace showing an overlay of a three seconds CO_2_ pulse (green bar) with 0.5 second methyl pyruvate pulse (red bar). **(B)** Dose-dependent inhibition of CO_2_-mediated activation of the *An. coluzzii* CO_2_ neuron over a range of five orders of magnitude. Chemical structures of the inhibitors are depicted on the right. n = 3. error = s.e.m.

Among the odorants that induced responses lower than the solvent, six odors actually inhibited the baseline activity of the *An. coluzzii* cpA neuron when tested with the headspace from 10^−2^ concentration odor cartridges (Fig. 1A). In order to test whether some of these odorants could constitute potential antagonists of cpA, we tested the ability of 21 odorants at a higher concentration (headspace from 10^−1^ concentration odor cartridges) to inhibit CO_2_-mediated (0.15% concentration) activation of the *An. coluzzii* cpA neuron in overlay assays. Of the 21 odorants tested, 11 were capable of reducing CO_2_-mediated cpA activation between 20% and 45%, and 5 odorants inhibited CO_2_ activation by >80% (Fig. 3A). Four top inhibitors were selected for dose-response assays. Four odorants were able to inhibit cpA activation by at least 50% when tested at 10^−2^ concentration and propanal evoked similar levels of inhibition when tested at 10^−3^ (Fig. 3B).

### Conservation of inhibitors in *An. sinensis*

**Figure 4:**
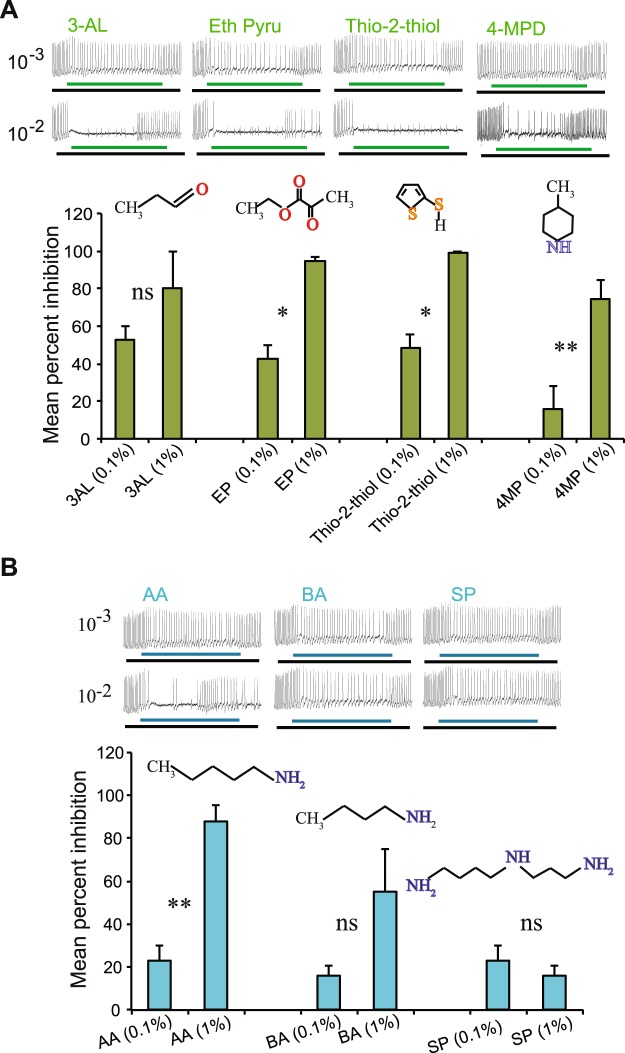
Inhibition of *An. sinensis* CO_2_ receptor neuron (cpA). **(A)** Representative traces, mean percent inhibition, and chemical structure of cpA background for propanal (3-AL), ethyl pyruvate (EP), thiophene-2-thiol (Thio-2-thiol) and 4- methyl piperidine (4-MP), and **(B**) amyl amine (AA), butyl amine (BA) and spermidine (SP). n = 6 sensilla. Green and blue bars indicate 0.5 s odor stimulus; black bars indicate the duration of the CO_2_ stimuli (1 s).

In order to test whether inhibitory odorants of the *An. coluzzii* cpA neuron could be of utility in *An. sinensis*, four of the strong inhibitors (propanal, ethyl pyruvate, thiophene-2-thiol, and 4-methyl piperidine) were tested at two concentrations using electrophysiology. All the tested odorants showed some degree of inhibition, but to varying extent. The strongest inhibition was observed for thio-2-thiol and ethyl pyruvate at the higher concentrations (1%) (Fig. 4A). Among the three tested amines, amyl amine (AA) exhibited the highest inhibitory activity followed by butyl amine (BA). Spermidine (SP), on the other hand, acted as a weaker inhibitor in the presence of CO_2_ (Fig. 4B). Taken together these results indicate that the *An. sinensis* CO_2_ receptors respond similarly to *Ae. aegypti* and *An. coluzzi* when it comes to CO_2_ response inhibition.

## Discussion

Unlike the complex blend of human skin odor, the CO_2_ in exhaled breath provides a simpler cue to study. Most mosquito species are strongly attracted to CO_2_ exhaled from human breath^2^. Carbon-dioxide is detected by the cpA neuron upon binding to a receptor, comprised of three members of the gustatory receptor gene family and named GR22, GR23, and GR24 in *An. coluzzii*^4,5^. The detection of CO_2_ plays several important roles in host-seeking behaviors like long-range navigation towards a live animal^2,14^. The identification of volatile ligands of the CO_2_ receptor neuron can contribute to the design of masking agents using inhibitors and trapping-lures using activators, both of which can reduce human contact and prevent disease transmission by mosquitoes^6^. We previously developed a computational approach^15^ which we applied to identify novel odorant ligands of the cpA neuron in *A. aegypti*^6^. Here we tested the conservation of the ligand responses in two species of anopheline mosquitoes, *An. coluzzii* and *An. sinensis*, that transmit malaria in Africa and Asia respectively. *An. sinensis* is one of the vectors of malaria in Asia and has Gr receptor orthologs that are closely related to ones in *An. coluzzi*. A high degree of functional conservation was observed amongst the ligands and we identified all three classes of ligands: activators, prolonged activators, and inhibitors.

In general, the responses to activators were weaker in *An. coluzzii* than in *Ae. aegypti*^6^, and only two odorants ((E)-pent-2-enal and methyl acetate) evoked stronger responses in *An. coluzzii* than in *Ae. aegypti*^6^. Conversely, inhibition of CO_2_-mediated cpA activation is stronger in *An. coluzzii*, as odorants unable to inhibit the cpA neuron in *Ae. aegypti*^6^ were capable of inhibiting the *An. coluzzii* and *An. sinensis* CO_2_ neuron counterparts. Amongst the inhibitors we have identified both high and low volatility compounds that act in both *An. coluzzii* and *An. sinensis*.

The detection of CO_2_ by the cpA neuron also activates attraction to other cues like skin odorants, visual cues, and importantly to body-warmth of 37 °C which is one of the strongest attraction cues at close-quarters^12,16,17^. The maxillary palp neuron, cpA also plays an important role in detection of human skin odorants^6^, and inhibitors such ethyl pyruvate and spermidine show reduction in attraction to skin odorants in *Aedes*^6,18^. Odorants like these could be useful in reducing host-seeking behavior and transmission of malaria, especially when used alongside others that block receptors detecting skin odorants.

Another approach to modulate behavior is using strong prolonged activators of cpA neurons that make the neuron unresponsive to CO_2_ as has been shown with 2,3-butanedione and blends on *An. coluzzii* and *Ae. aegypti*^13^. However, at the higher concentration needed for this effect the unpleasant smell of this odorant and health concerns precluded integration into practical solutions. We were able to demonstrate that *An. coluzzii* showed an ultra-prolonged activation to (E)-2-methylbut-2-enal, which resulted in masking the detection of CO_2_ significantly for several minutes after by the maxillary palp cpA neurons suggesting that this odor could disrupt detection of CO_2_ and navigation toward its source as has been shown in *Ae. aegypti*^13^ and could be utilized for potential practical applications in preventing mosquito bites and spreading of mosquito-borne diseases^19^.

Some of the CO_2_ receptor neuron inhibitors have organoleptic and physicochemical properties that are conducive to development into spatial and short-range masking agents for anopheline mosquitoes. However, ultimately for a masking strategy to work, additional odorants will be needed in a blend to block other human skin receptors that are members of the *Odorant receptor (Or)* and *Ionotropic receptor (Ir)* gene families.

## Experimental Procedures

### Mosquitoes

The M form of *Anopheles gambiae* (Herein *An. coluzzii*, Ngousso strain, Cameroon) were maintained in a 12:12 (Light:Dark) photocycle at 27 °C and 70% RH. The *An. sinensis* mosquitoes were received from MR4 center and then colony was maintained in insectary at the same conditions as *An. coluzzii*. Adult females were fed on bovine blood through a heated membrane feeding system (Hemostat Laboratories, California, USA).

### Electrophysiology

Single-sensillum recording was carried out with 4–12 days old female anopheline mosquitoes as described elsewhere^6,13,20^. All recording replicates were performed in different specimens. All odorants were obtained from Sigma at >98% purity and were diluted to (as indicated) in paraffin oil or water. A filter paper with 50 μl of the solution was inserted into a Pasteur pipette cartridge and the headspace was injected into a humidified airstream to further dilute it 3-fold as done previously^6^.

For the ultra-prolonged activators (E)-2-methylbut-2-enal, 3-methylbut-2-enal and 3-methylbutanal were dissolved at 10^**−**1^ in paraffin oil or water, from which 50 μl of the solution was added on a filter paper inside a Pasteur pipette and the headspace was used for odor delivery as indicated in the section above. The odor delivery system was modified as shown in^6^; solvent responses during the same recording session were subtracted. A controlled 3-s stimulus of solvent/stimulus was delivered from a Pasteur pipette into the carrier airstream. Subsequent 1-s of CO_2_ (0.15%) stimuli was delivered using a MNJ-D microinjector (Tritech Research). Activity was calculated by subtracting baseline activity 1-s prior to each stimulus. Spike counting was done with Clampfit 10.3.

### Phylogenetic analysis

The amino acid sequences of the CO_2_ receptor orthologs of *Drosophila melanogaster*, *An. coluzzii*, *An. sinensis*, *Ae. aegypti*, and *Cx. quinquefasciatus* (signal peptide removed) were aligned with the ClustalW software, and the phylogenetic tree was constructed with the MEGA6^21^ software, using the Maximum Likelihood method and LG + G matrix-based model^22^. Reliability of the branches was inferred by 1,000 bootstrap replicates^23^.

### Sequence access numbers

The GenBank access numbers for the CO_2_ receptor are as followed: *An. coluzzii* GR22 (XP_319142), GR23 (XP_312786), and GR24 (ABK97614); *An. sinensis* GR22 (KFB40998), GR23 (KFB38231), and GR24 (KFB40736); *Ae. aegypti* GR1 (XP_001655150), GR2 (XP_001654839), and GR3 (XP_001660602); *Cx. quinquefasciatus* GR1 (XP_001848097), GR2 (XP_001848828), and GR3 (XP_001848689); and *Drosophila melanogaster* GR21a (ABK97615) and GR63a (ABK97613).
